# Editorial: Highlights of POG 2019 - Plant Oxygen Group Conference

**DOI:** 10.3389/fpls.2021.639262

**Published:** 2021-02-01

**Authors:** Christian Lindermayr, Krystyna Oracz, Ann Cuypers, Jörg-Peter Schnitzler, Jörg Durner

**Affiliations:** ^1^Institute of Biochemical Plant Pathology, Helmholtz Center Munich, Neuherberg, Germany; ^2^Department of Plant Physiology, Institute of Biology, Warsaw University of Life Sciences, Warsaw, Poland; ^3^Center for Environmental Sciences, Hasselt University, Diepenbeek, Belgium; ^4^Research Unit Environmental Simulation, Institute of Biochemical Plant Pathology, Helmholtz Center Munich, Neuherberg, Germany; ^5^Chair of Biochemical Plant Pathology, Technische Universität München, Freising, Germany

**Keywords:** reactive oxygen species, reactive nitrogen species, redox-signaling, S-sulfenylation, S-nitrosation, nitration

Redox reactions are evolutionarily conserved signaling principles, occurring in prokaryotes and eukaryotes. The most important redox molecules are reactive oxygen and nitrogen species (ROS and RNS). Due to their short half-life, high diffusion capability and ability to react with different components in the cell, ROS, and RNS are key signaling molecules participating in various signaling pathways involved in the regulation of transpiration, gas exchange, biotic/abiotic stress response, cell death, germination, and plant growth and development (Del Río, 2015; Mhamdi and Van Breusegem, 2018; Waszczak et al., 2018; Sánchez-Vicente et al., 2019; Smirnoff and Arnaud, 2019; Sharma et al., 2020). In detail, they modify proteins and cellular metabolites and in this way alter their activity, function, stability, and/or intracellular localization (Mata-Pérez et al., 2017; Mittler, 2017; Czarnocka and Karpinski, 2018; Umbreen et al., 2018; Gupta et al., 2020). This Research Topic introduces active research in this rapidly moving field, including functional analysis of these redox-active molecules as well as technical developments in redox research.

## Redox-Signaling Mechanisms

Modification of biomolecules, such as proteins, lipids, or nucleic acids, by ROS and RNS represents one of the key mechanisms mediating the biological activity of these redox molecules. Petrivalský and Luhová discuss in their mini-review article the current knowledge on formation, metabolism and biological function of nitrated nucleotides focusing mainlsy on 8-nitroguanosine 3′,5′-cyclic monophosphate (8-nitro-cGMP). 8-nitro-cGMP is formed by the reaction of peroxynitrite with free guanine nucleotide and possess the strongest redox-active and electrophilic properties among studied nitrated guanine derivatives. The signaling function of 8-nitro-cGMP is based on one side on its similarity to cGMP and on the other side on its electrophilic features and the chemical interaction with protein thiol groups resulting in the formation of a novel post-translational modification of cysteine residues termed S-guanylation. In plants, until now a physiological function of 8-nitro-cGMP has only been described in *Arabidopsis thaliana* stomatal guard cells, thus, it still requires intensive investigation to unravel mechanisms and biochemical and physiological functions of 8-nitro-cGMP and protein S-guanylation.

S-Sulfenylation is an oxidative modification of cysteine residues that can alter protein interaction, trafficking, conformation, and enzymatic activity. Wei et al. present an advanced non-invasive method for identifying sulfenylated cysteine residues. The method is based on the redox-sensitive cysteine residue Cys598 of the yeast AP-1-like transcription factor, which specifically reacts with sulfenic acids resulting in the formation of a mixed disulfide bond. After tryptic digestion, the mixed disulfide-linked peptides can be enriched using an antibody against the Cys598-containing peptide. The presented novel labeling approach enables the identification of sulfenylated cysteines in any species that can be genetically modified and allows a deeper insight into the signaling function of S-sulfenylation.

## Redox-Regulation of Plant Growth and Development

Redox-dependent control of plant growth and development involves a network of interactions between redox-active molecules, antioxidants, and phytohormones. ROS are essential regulators of fruit ripening. Gonzalez-Gordo et al. focused on the superoxide metabolism during the non-climacteric sweet pepper ripening and revealed that superoxide generating NADPH oxidases accumulated during ripening. This superoxide generating system is modulated in a NO-enriched environment. Interestingly, the activity of different superoxide dismutase isoenzymes was unaffected during ripening suggesting that the basal superoxide dismutase activity is sufficient to keep the homeostasis of the necessary physiological superoxide production during sweet pepper ripening. Besides the importance for the ripening process, the superoxide production could also have additional benefits for the fruits as barrier preventing potential pathogen infections.

Class III plant peroxidases are involved in the oxidative polymerization of lignin. Comprehensive understanding of the lignification process has significant impact on industrial biofuel production, wood quality, and biodegradable plastic production. García-Ulloa et al. overexpressed *Zinnia elegans* basic peroxidase (ZePrx) in *Nicotiana tabacum* to further characterize its function in lignin biosynthesis and its interconnection with the antioxidative system *in planta*. According to the presented results, ZePrx functions in secondary cell wall biosynthesis by increasing sinapyl lignin in cell wall stems. Furthermore, increased ascorbate peroxidase activity and a reduced ascorbate redox state was observed confirming a role of ZePrx in maintaining the redox homeostasis.

## Redox Control of Plant Stress Response

Plants are subjected to various abiotic and biotic stresses throughout their life cycle. Redox species play important functions in enabling stress tolerance. To optimize photosynthetic and acclimatory processes, regulation of light absorption under variable light conditions is essential. Czarnocka et al. identified new functions of the J domain-containing protein required for chloroplast accumulation response 1 (JAC1). So far, this protein was described in context of chloroplast movement. However, the presented results demonstrate pleiotropic functions related to regulation of photosynthetic reactions, redox homeostasis, and cell death.

In plants, dioxygenases perform a variety of functions ranging from hormone biosynthesis to epigenetic rearrangements of chromatin. In their review, Iacopino and Licausi summarize the state of knowledge on the most important signaling functions of dioxygenases in plants. The focus of their article is on the importance of dioxygenases in adaptive processes under oxygen-deficient conditions, such as flooding. Due to their need for co-substrates and co-factors, such as oxygen, 2-oxoglutarate and iron (Fe^2+^), the authors propose a potential role of dioxygenases as metal sensors in plants.

Alternative oxidase (AOX) is a terminal oxidase in the plant's mitochondrial electron transport chain, which is not contributing to ATP synthesis. Jayawardhane et al. investigated the roles of AOX under different oxygen conditions using AOX overexpressing and knockdown *N. tabacum* plants. Interestingly, they observed the accumulation of NO under hypoxia and hypothesized that AOX is able to generate NO under hypoxia or that AOX activity reduces the amount of the NO scavenger superoxide. Overall, they demonstrated that AOX fulfills beneficial functions in low oxygen metabolism.

Cadmium (Cd) exposure causes an oxidative challenge and inhibits cell cycle progression. Such stress-induced effects on the cell cycle are often a consequence of activation of the DNA damage response (DDR). Hendrix et al. reported the involvement of Suppressor of Gamma Response 1 (SOG1) in the Cd-induced DDR in Arabidopsis leaves. Additionally, they described a new function for SOG1 in regulating the oxidative stress response in Cd-exposed plants. Hendrix et al. proposed that SOG1 might function as a general integrator of stress responses in plants.

Plants growing in mining areas can accumulate high levels of antimony (Sb) in their edible parts, causing potential health risks for herbivores and also for humans. In this respect, understanding the mechanisms involved in the uptake, toxicity, and detoxification of Sb is of great importance. Espinosa-Vellarino et al. analyzed the effects of Sb toxicity on plant growth, ROS/RNS, and the antioxidant systems in tomatoes. They show that Sb accumulation in roots impairs micro-elements uptake, plant growth, and photosynthetic performance. In response to Sb uptake, an increased production of reactive oxygen and nitrogen compounds occurs. Moreover, they demonstrate that this is accompanied by a strong expression of enzymes of the antioxidative system and causes tissue-specific changes in the levels and redox status of antioxidants. All this seems to be related to the ability of Sb to form complexes with thiol groups, which changes the redox homeostasis of plants.

## Impact of Redox-Active Atmospheric Gases on Plants

Industrial processes, residential heating, and heavy traffic based on fossil fuels result in high levels of particles, nitrogen oxides (NOx), and other dangerous volatile organic compounds (VOCs) in urban and suburban areas. Since especially long-term exposure to such harmful particles/gases can negatively affect human health, it is desirable to lower their concentration in cities. Zhang et al. determined the NO and NO_2_ specific deposition velocities based on projected leaf area using a branch enclosure system and NO uptake in ^15^NO fumigation experiments. They studied tree species that are regarded as suitable to be planted under predicted future urban climate conditions. Their results demonstrate that selection of appropriate tree species able to cope with increased heat and drought stress while keeping a high capacity to “clean” air may thus support urban planning strategies.

Plants are masters in adapting quickly and efficiently to changing environmental conditions. The expected increase in atmospheric CO_2_ content will have a strong impact on plants, especially those that perform C3 photosynthesis. To develop sustainable management practices for agricultural crop protection, it is therefore crucial to understand how increased CO_2_ levels affect plant-environment interactions, especially biotic stress caused by herbivores or microbial/fungal pathogens. In their work, Martinez Henao et al. applied a multifactorial design to analyze the effects of different N-fertilization (nitrate and ammonium) at ambient or elevated CO_2_ concentrations on the response of *A. thaliana* plants to artificial injury, simulating an attack by herbivores or a necrotrophic pathogen. They show that artificial wounding leads to an increased oxidative status and to the accumulation of jasmonate phytohormones. The plant response to wounding, which depends on the type of fertilizer, is however, alleviated in plants grown under an increased CO_2_ content. With this work the authors shows that a deeper understanding of the mechanisms underlying plant defense is crucial for the development of strategies under future predicted climatic conditions.

## Concluding Remarks

The collection of 11 research and review articles in this Research Topic reflects the broad spectrum of current research directions in the field of redox biology—from general plant cell metabolism, plant growth, and development, to abiotic and biotic stress effects and the identification of new antioxidant defense mechanisms. Plants produce ROS and RNS during growth/development and stress response. As the contributions to the RT “Highlights of POG 2019 - Plant Oxygen Group Conference” exemplarily shows, the redox-active molecules fulfill important signaling functions via various protein modifications, such as S-nitrosation, tyrosine nitration, or S-sulfenylation. Furthermore, nitrated nucleotides and functions of dioxygenases seem to be new players in ROS and RNS signaling. Depending on the stage of development or environmental stressors, different redox processes are induced in different cellular compartments, which proves the existence of a highly coordinated “redox network” in plants. Interestingly, atmospheric redox-active gases such as NOx can also disturb the cellular redox processes and influence plant growth/development and stress response. The work collected in this RT impressively demonstrates that research on reactive oxygen and nitrogen species remains central to a holistic understanding of the adaptation mechanisms of plants to their abiotic and living environment and their underlying regulatory networks.

## Author Contributions

CL and J-PS wrote the initial draft of the Editorial. KO, AC, and JD made final comments. All authors contributed to the article and approved the submitted version.

## Conflict of Interest

The authors declare that the research was conducted in the absence of any commercial or financial relationships that could be construed as a potential conflict of interest.
